# A scoping review of the research literature on eating and body image for transgender and nonbinary adults

**DOI:** 10.1186/s40337-023-00828-6

**Published:** 2023-07-04

**Authors:** Katie Heiden-Rootes, Whitney Linsenmeyer, Samantha Levine, Mark Oliveras, Miriam Joseph

**Affiliations:** 1 Department of Family and Community Medicine, School of Medicine, Saint Louis University, 3700 Lindell Blvd., Ste 1100, St. Louis, MO 63108 USA; 2 Department of Nutrition and Dietetics, College of Health Sciences, Saint Louis University, St. Louis, MO USA; 3 University Libraries, Saint Louis University, St. Louis, MO USA

**Keywords:** Eating, Body image, Disordered eating, Weight, Transgender

## Abstract

**Background:**

Eating disorder treatment approaches and outcome studies have historically centered almost exclusively on cisgender populations. Transgender and nonbinary (TGNB) adults are underrepresented in general and intervention research despite being at increased risk for eating and body image-related problems.

**Aims:**

This scoping review was designed to gather and examine the research with TGNB adults who experience eating and body image related problems, as well as clinical studies on the effectiveness of treatment approaches.

**Method:**

Preferred Reporting Items for Systematic Reviews and Meta-Analyses extension for Scoping Reviews (PRISMA-ScR) was used for reporting this review. MEDLINE and PsychInfo were used as electronic databases for searching subject terms. Inclusion criteria for studies required the quantitative measurement or qualitative exploration of body image or eating for TGNB adults. The relevant data were extracted and summarized based on quantitative findings and qualitative themes.

**Results:**

After review of over 1258 articles, 59 studies met criteria and data were extracted and summarized. Factors associated with eating disorders and body image problems across studies suggests gender-affirming medical interventions are effective and emphasized treatment for an eating disorder is warranted alongside gender affirming medical care. Body image was associated with eating patterns aimed at meeting gendered ideals of body shape and size. There was variation in guiding theories and absence of consensus in the definition of transgender in the review studies. This likely demonstrates the changing language, social acceptance of TGNB people and identities, diagnostic criteria, and clinical conceptualizations of eating and body image.

**Conclusions:**

Future research should consider the use of theory for guiding inclusion of salient social factors influencing eating patterns, body image, and treatment outcomes. In addition, future research is needed that centers on nonbinary and genderqueer populations, as well as those from minoritized racial and ethnic groups to inform culturally appropriate concerns, needs, and treatment modalities.

## Abstract (plain language summary)

Fifty-nine published research studies with transgender adults on eating patterns, body image, and associated risks and comorbidities were collected and summarized. Across studies, gender affirming medical interventions like hormone therapy and surgery were associated with decreases in eating disorder symptoms and improved body image. Studies from interviews with transgender adults found social causes for disordered eating and poor body image including rejection and discrimination. Future studies should use guiding theories for testing the causality and consider rejection and discrimination experienced by transgender adults.

## Background

Eating disorder treatment approaches and outcome studies have centered almost exclusively on cisgender (i.e., sex-assigned-at-birth aligns with gender identity) participants [1]. Transgender and nonbinary (TGNB; i.e., gender identity differs from sex-assigned-at-birth, inclusive of transmasculine, transfeminine, gender queer, and other genders) adults are underrepresented in research despite disproportionate prevalence rates of eating disorders and body image problems [2]. Gender dysphoria is associated with disordered eating, body image problems [3], and minority stress [4]. This may explain the higher prevalence rates of eating disorders and body image problems for TGNB people [2] and the need for innovations in treatment and intervention.

### Minority stress: a guiding theory

A guiding theory for this review is Minority Stress Theory (MST) as it has been applied and expanded for TGNB people. MST, first theorized in the context of gay, lesbian, and bisexual people [5], posits there are external and internal stressors acting on the health and safety of TGNB adults [6]. External stressors include stigma, rejection, employment and housing discrimination, and violence experienced because someone is transgender. External stressors create internal stressors of self-rejection (i.e., internalized transphobia), increased gender dysphoria (e.g., psychological distress associated with gender identity not matching the body/sex-assigned-at-birth), expectations for rejections by others, and pressure to conceal transgender identity and gender expression [7]. Increased external and internal stressors, as MST posited [6], lead to poor physical and mental health [8] and eating and body image problems [4].

MST also proposes key protective factors for TGNB adults, including social acceptance in family, work, social, and healthcare settings (e.g., use of correct pronouns, name, and maintaining a relationship after disclosure of transgender identity), social changes (e.g., name and pronoun changes, gender marker), and access to medical services (e.g., puberty blockers, hormones, surgery, affirming psychotherapy and treatment) [6]. Acceptance and support are protective factors because they may reduce and, in some cases, eliminate gender dysphoria for TGNB adults [9]. Unsurprisingly, these lead to improvements in both mental health [10] and eating and body image-related problems [11]. There is a growing body of research to support using MST to explain differences in the health outcomes of TGNB people, which includes efforts to consider the compounding impact of multiple marginalized identities for TGNB people from minoritized ethnic and racial communities [12].

MST for TGNB research and in this scoping review considers psychological and social factors as significant contributors to outcomes of marginalized subgroups within the TGNB population. Thus, MST aids in identifying missing factors and subgroups (e.g., TGNB people from marginalized racial and ethnic groups) not included in the current literature body. It also provides a critical lens to the review analysis about the types of methods employed for studying TGNB experiences, the language used about TGNB patients, and the treatment approach and outcomes of TGNB people in healthcare. External stressors are experienced across social settings for TGNB people, including in healthcare and research participation, impacting their health and safety [13], thus MST guides this review to take a reflexive look at what empirical research is saying about TGNB adults.

### Current study

MST posits that TGNB health outcomes will vary based on external and internal stressors, which are further influenced by mental health comorbidities, age, social acceptance, race/ethnic group, and access to gender affirming healthcare and treatment. MST offers a lens for examining the literature with TGNB adults with eating and body image related problems. As a result, this scoping review aimed to answer four research questions: 1) What methodologies are being used to study eating and body image related problems with TGNB people? 2) What are the risks and protective factors for eating and body image related problems? 3) Who is being included and excluded in the TGNB samples of studies on eating and body image related problems? 4) What are the empirically supported treatments for eating and body image problems for TGNB patients? The focus of this paper is on adults only. Adolescent and young adult literature is detailed in another scoping review (Authors, et al., under review).

Current analyses that center TGNB people are narrative in nature [14, 15], span broader sexual and gender minority populations [16], or provide a narrowed focus on diagnosis rates and symptom presentation [16–18]. To inform continued research and treatment innovations, we conducted a scoping literature review to critically examine research about TGNB adults who experience eating and body image problems and any clinical studies that detail treatment approaches and their effectiveness. This review expands on current analyses by incorporating studies that address eating disorder treatments and interventions, mental health comorbidities and gender dysphoria, and general eating patterns that are not necessarily disordered in nature. We also sought to include articles at the intersection of eating and body image given the theorized utility of disordered eating behaviors to attain a body size or shape that is an attempt to meet gendered appearance ideals. In this way, the scoping review maps the breadth of the literature and identifies gaps in the knowledge base [19].

## Method

This literature review adhered to the Preferred Reporting Items for Systematic Reviews and Meta-Analyses extension for Scoping Reviews (PRISMA-ScR) guidelines in the search, review, and reporting processes [20, 21]. The search strategy was developed through initial meetings and consultation between the first author and the university librarian (last author, MJ) in the fall of 2020. Preliminary searches were conducted using the OVID interface of possible databases including *MEDLINE, PsychINFO, CINAHL: Cumulative Index to Nursing and Allied Health Literature, Cochrane Database of Systemic Reviews, Social work Abstracts, Social Services Abstracts,* and *Sociological Abstracts* to identify potential articles about transgender adults and eating, body, and weight related problems. The second author (WL) was consulted based on her expertise to review initial searches for relevant articles. The preliminary searches demonstrated two databases—*MEDLINE* and *PsychINFO*– were superior for identifying relevant articles. To search the databases, the OVID interface was used, and official subject terms were identified for each database to ensure the use of consistent vocabulary. In MEDLINE, the search terms—*transgender persons, gender identity, transsexualism, gender dysphoria, body image, body dissatisfaction, self-concept, feeding behavior, anorexia nervosa, binge eating,* and *bulimia nervosa*—were used. The trans/gender terms and body/eating terms were then searched together for identifying articles where both subjects were categorized*.* A similar process was used for PsychINFO with the following subject terms—*transgender, gender dysphoria, gender identity, gender nonconforming, transsexualism, body image, body esteem, body satisfaction, body dissatisfaction, body awareness,* and *eating behavior or attitudes or disorders. Eating behavior or attitudes or disorders* subject terms included anorexia, bulimia nervosa, and binge eating disorders. No limits were set by date of publication in order to capture the changing theories and findings in the field up to literature published online or in print in December 2022.

### Inclusion and exclusion criteria

Articles included in this review met the following criteria: 1) published in peer review journals (including online advance publications); 2) published in English language by December 2022; 3) described qualitative or quantitative empirical research (including case reports and case studies); 4) addressed review questions about eating behavior and body image including those addressing treatment and intervention; and 5) sample of transgender participants (inclusive of transgender men, women, nonbinary, and gender expansive or questioning) over the age of 18 years old. The following types of studies were excluded from the review: book chapters; review articles; editorial commentaries; clinical opinion articles without case or research data; non-English language studies; dissertations; studies where outcomes from transgender participant data were not reported separately from larger sample; and studies that did not include at least one of the following—eating behavior or disorder measurement, body image measurement, or interview data on eating or body image. Finally, we removed studies examining only young adults samples (ages 18 to 25) as a unique part of adolescent development [22]. These samples are included in our youth/young adult scoping review article (Authors et al., under review).

### Review procedure and analysis

**Fig. 1:**
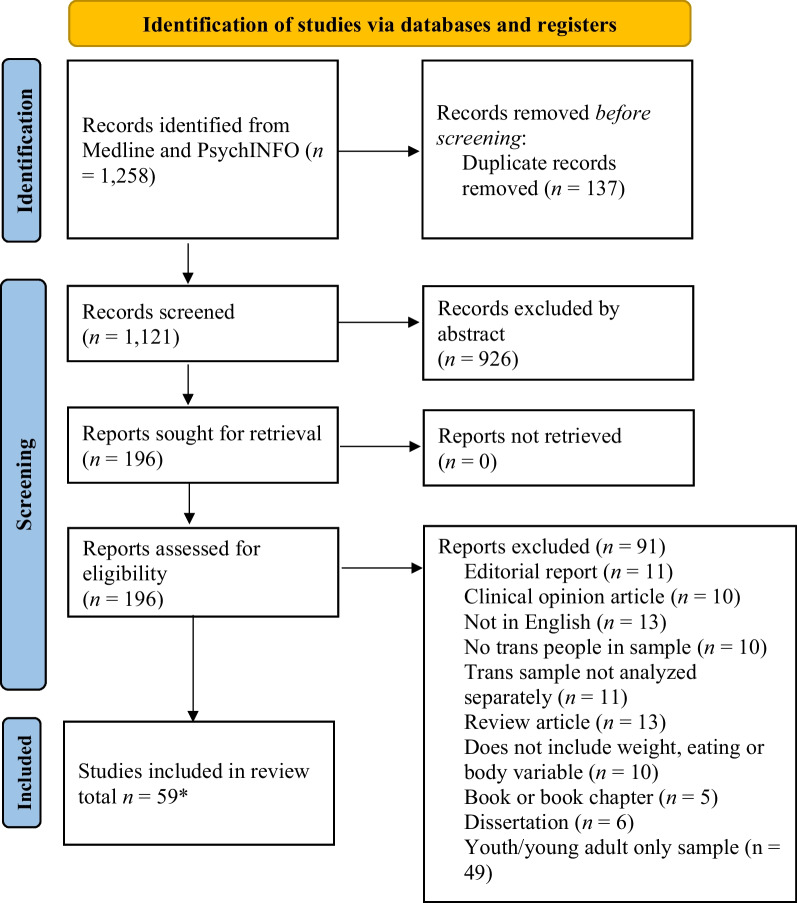
PRISMA 2020 Flow Diagram of the systematic literature review process. *Note. Some samples included both youth and adults with analysis by age for obtaining separate results based on age/developmental period

The identified articles were uploaded to Covidence©, an online software, for managing duplicate removal and then the processes of abstract review, full text review, and, finally, data extraction. Duplications were removed initially by the software. This was reviewed by the first author to ensure accuracy of the removal. Reviews were completed by three research team members (KHR, WL, SL) and four graduate research assistants. Pairs of authors and graduate research assistants reviewed abstracts based on the inclusion/exclusion criteria. Discrepancies in the reviews were resolved by the first and second authors. Then full text reviews were completed by the second and third authors and the graduate research assistants. Again, discrepancies were resolved by the first and second authors. Data extraction was completed by the second, third and fourth authors. Finally, one case control study article was removed at data extraction as two reviewers (KHR, SL) agreed that the study did not meet our inclusion criteria as transgender people were the subject of the work, but not the participants in the study [23]. The PRISMA figure (see Fig. 1) outlines the course of the review and article selection and extraction.

Data were finally downloaded from Covidence© to a spreadsheet developed by the first author where the sample characteristics, guiding theories, definitions of ‘transgender’ and/or language about gender, measurements, funding sources, limitations, bias, and other commentary were noted. The third and fourth authors led the data movement to the spreadsheet. Then analysis of the data followed three modalities. First, studies were separated by age to create youth (children to young adult where young adults were analyzed as unique developmental stage) and adult (adult-only samples, 18 years old and older with broad age ranges and analyzed together as an all-adult sample) tables for separate analysis. Again, this manuscript will only report on the adult samples and the youth and young adult articles are summarized in a separate scoping review manuscript (Heiden-Rootes et al. [24]).

Then, study methodology (quantitative, qualitative, and case report) was analyzed by the first and third authors. The method of articles was analyzed for identifying and quantifying types of methodology, sample size and demographics, measurement use, and geographic location. We sought to summarize and critique the current body of literature based on method and the limitations this may create for drawing conclusions and implications from the studies. Finally, variable outcomes (eating disorders, body image) were analyzed by the second and fourth authors. Variable outcomes were reported based on divergent and consistent findings across studies. In addition, unique findings were noted for subsamples, if appropriate. The outcome results were then narratively summarized.

To assess rigor and potential bias in studies, the authors used four items from the STrengthening the Reporting of OBservational Studies in Epidemiology (STROBE) checklist [25] and the Standards for Reporting Qualitative Research (SRQR) checklist [26] as previously used in systematic literature reviews with women with minoritized sexual identities [27]. This allowed for a critical review of study biases. The items were: (1) The authors describe the eligibility criteria and the sources, methods, and rationale of participant selection; (2) The authors describe the characteristics of study participants (coded “yes” if the authors provided information about age, race/ethnicity, and socioeconomic status); (3) The authors describe and provide a rationale for their quantitative or qualitative analytic methods; and (4) The authors discuss the limitations of the study, including sources of potential bias or imprecision. This allowed for the potential for bias to be assessed by the first and second author after data extraction of the results was completed. Finally, funding source was noted for gaging the degree of funding available for TGNB studies on eating and body image and for identifying entities who were investing in research with and for TGNB adults.

## Results

The review identified 59 articles for inclusion (see Table 1 for PRISMA figure and Table 2 for Synthesis of Results by Research Question). The results section is organized by areas of analysis to answer the five research questions—bias and limitations, method analysis (including sample demographics and geographic location of studies), and summary results by topic area (eating problems, body image).

**Table 1:** Final adults articles retained for review

References	Sample size	Eating Disorder or Body Image focus	Sample demographics	Study design	Quantitative measures	Qualitative interview domains	Findings	Country of origin
Algars et al. [28]	n = 21	Eating Disorder	11 adult transgender men participants and 9 adult transgender women participants	Qualitative interviews with brief survey	Body Mass Index and EDI-3 survey	Open ended questions such as "tell me about your relationship to food and eating", "describe what you eat during a typical day in your life" etc	A majority of both trans men and trans women participants reported past or current disordered eating. Attempts to suppress biological gender or accentuate desired gender most often drove the desire for thinness	Finland
Algars et al. [29]	n = 1,142	Body Image and Disordered Eating	698 adult women and 444 adult men	Cross-sectional survey	Derogatis Sexual Functioning Inventory body image scale, Eating Attitudes Test, Gender Identity Scale	N/A	Those with conflicted gender identity showed higher levels of body dissatisfaction. Women with a conflicted gender identity also showed more eating disturbance than controls	Finland
Auer et al. [30]	n = 154	Body Image	82 adult transgender women and 72 adult transgender men	Cross-sectional survey	Short-form Health Survey, Beck Depression Inventory II, Social Support Scale, Fragebogen zur Beuteilung des eigenen Korpers State-Trait Anxiety Inventory Form X, Pittsburgh Sleep Quality Index, The German pain questionnaire	N/A	Insecurities about appearance and poor self-perception of body image was an independent predictor for quality of life for transgender women but not for transgender men	Germany
Bandini et al. [31]	n = 295	Body image and eating disorders	50 adult trans women participants, 50 adult trans men participants	Cross-sectional survey, Between groups comparisons	Symptom Checklist, Body Uneasiness Test	Structured Clinical Interview for Diagnostic and Statistical Manual of Mental Disorders, Fourth Edition	Transgender participants without genital reassignment surgery had higher levels of body uneasiness compared other groups. Trans women showed higher values than both controls and eating disorder subjects in the compulsive body self-monitoring domain, while no differences were observed between trans men and controls in this area	Italy
Bandini et al. [32]	n = 109	Body image	Adults	Cross-sectional	Body Uneasiness Test; Symptom Checklist-90 Revised; 19-item yes/no checklist covered childhood maltreatment and family relationships, gender identity and SRS, sexual orientation, and psychiatric history	Patients were asked to specify any current pharmacological treatment	Childhood maltreatment was associated with a higher level of body image dissatisfaction and worse lifetime mental health. Participants who reported childhood maltreatment reported higher body compulsive self monitoring and worry about certain body parts	Italy
Becker et al. [9]	n = 202	Body image	62 adolescent trans women, 20 adolescent trans women, 50 adult trans men, and 70 adult trans women	Cross-sectional	Body Image Assessment Questionnaire: “attractiveness/self-confidence” scale, “accentuation of body appearance” scale, “insecurity/concern” scale, and “sexual-physical discomfort” scale	N/A	Adolescents had a less favorable body image compared to adults on all four scales. Transgender participants who already received medical interventions reported a less impaired body image	Germany
Bell et al. [33]	n = 317	Eating disorders	97 gay adult men, 82 lesbian adult women, and 138 TGNC adults Racial diversity present	Cross-sectional	Patient Health Questionnaire Depression Scale, Generalized Anxiety Disorder 7, Self-Compassion Scale- Short Form, Negative Social Exchange Subscale of the Multidimensional Health Profile: Psychological Functioning, Interpersonal Needs Questionnaire, Perceived Stigma Scale; National College Health Assessment, Eating Disorders Screen for Primary Care	N/A	Lesbian women and TGNC adults were more likely than gay men to report current or past experience with an eating disorder, and TGNC adults were more likely to report dissatisfaction with eating patterns. Self-compassion was inversely associated with ED proneness among TGNC adults	Australia
Bozkurt et al. [34]	N = 160	Body image	36 gay adults, 52 trans women adults, and 72 controls	Cross-sectional	Body Cathexis Scale; Eysenck Personality Questionnaire	N/A	The trans women participants were more satisfied with their waist, hips, eye shape, and weight compared to control participants, as well as height, legs and body postures, but were not satisfied with their shoulder width, arm length, or genitals	Turkey
Brewster et al. [4]	N = 205	Body image and Eating Disorders	205 transgender adult women. Racial diversity present	Cross-sectional	Heterosexist Harassment, Rejection, and Discrimination Scale; Interpersonal Sexual Objectification Scale; internalization general subscale of the Sociocultural Attitudes Toward Attractiveness Questionnaire-3; body surveillance subscale of the Objectified Body Consciousness Scale; Body Image Ideals Questionnaire; Eating Attitudes Test	N/A	Dehumanization was found to have a positive relationship with internalization and a positive direct and indirect relationship with disordered eating. Internalization was found to have a positive relationship with body surveillance, body dissatisfaction, and a direct and indirect relationship with disordered eating	United States
Carretta et al. [35]	N = 192	Eating disorders and body image	192 adult drag queens, Racial diversity present	Cross-sectional	Drag Queen Performance Style Scale developed for this study, Internalization (general) subscale of the Sociocultural Attitudes Toward Appearance Questionnaire-3, Upward Comparison subscale of Upward and Downward Appearance Comparison Scale, Centrality subscale of the In-Group Identification Scale, Eating Attitudes Test, Acceptance of Cosmetic Surgery Scale	N/A	Hyper-feminine drag was positively associated with disordered eating while gender-fluid drag was not. Further, this relationship between disordered eating and hyper-feminine drag is mediated by both internalization of cultural standards of beauty and upward appearance comparison, but was not mediated by identity salience	United States
Case et al. [36]	N = 16	Body image	8 trans men and 8 cisgender women adults	Cross-sectional	Somatosensory evoked fields recorded via magneto-encephalographic scanner followed by diffusion tensor imagining	N/A	Trans men participants demonstrated differences in neural processing of incongruent-feeling body parts between transgender participants and controls. Trans men participants on testosterone therapy rated their pre-op chests as belonging to them more than those not on hormone therapy, however this difference was not demonstrated on MEG imaging	United States
Castellano et al. [37]	N = 120	Body image	46 transgender adult women, 14 transgender adult men, 45 cisgender women, and 15 cisgender men	Cross-sectional	Quality of Life general score, Quality of sexual life subscore, quality of body image subscore, and hormone levels	N/A	The transgender group and control group did not differ in quality of body image or in quality of life. In addition, there was an inverse relationship between LH levels and both body image score and quality of sexual life score, while there was a non-significant relationship between quality of life and LH levels	Italy
Cella et al. [38]	N = 325	Body image and eating disorders	85 gay adult men, 47 gay women, 89 heterosexual men, 89 heterosexual women, and 15 TGNB adults	Cross-sectional	The Eating Disorders Inventory-2; the Eating Disorders Inventory-2-Symptom Checklist; The Body Uneasiness Test	N/A	Transgender participants reported higher levels of eating pathology and higher frequency of weight gain compensation strategies compared to the other study groups. Feminine participants score higher than masculine participants on drive for thinness, bulimia, body dissatisfaction, weight phobia, and body image concern	Italy
Chivers and Bailey [39]	N = 39	Body image	21 trans men gay participants and 17 non-gay trans men participants Racial diversity present	Cross-sectional	Modified Kinsey scale, The Childhood Gender Nonconformity scale, The Continuous Gender Identity scale, The Preference for Partner Masculinity scale Items for scales assessing concern with partner status, partner attractiveness, and youth, The Sexual vs. Emotional Jealousy Scale, The Passive Sexual Role scale, Interest in Uncommitted Sex and Interest in Visual Sexual Stimuli scales, and the Body Modification Scale	N/A	Higher cross-gender identification in childhood was correlated with a stronger interest in masculinizing body modification among trans men participants, with a greater desire for phalloplasty among homosexual trans men participants than non homosexual trans men participants. Continuous Gender Identity was related to desire for phalloplasty, but Childhood Gender Non-conformity was not	United States
Comiskey et al. [40]	N = 173	Body image and Disordered Eating	173 transgender women Adults, racial diversity present	Cross-sectional	Sociocultural Attitudes Toward Appearance Questionnaire Internalization subscale, Transgender Congruence Scale Appearance Congruence subscale, Body surveillance subscale of the Objectified Body Consciousness Scale; Body Shame subscale of the OBCS; Eating Attitudes Test; Anabolic–Androgenic Steroids measure to assess for intent to use silicone injections	N/A	Internalization of cultural standards of appearance was positively associated with body surveillance, which was positively associated with body shame. Body shame was positively associated with disordered eating and intention to obtain silicone injections	United States
de Vries et al. [41]	N = 55	Body image	22 transgender women and 33 transgender men Age diversity present	Prospective cohort	Utrecht Gender Dysphoria Scale (UGDS), Body Image Scale (BIS), Children's Global Assessment Scale, Beck Depression Inventory, Spielberg’s Trait Anger Inventory, Spielberg’s Trait Anxiety Inventory, Child/Adult Behavior Checklist, Youth/Adult Self-Report, a questionnaire created for this study used to ask the young adults about their current life circumstances, such as living conditions, school and employment, and social support, WHOQOL-BREF, Satisfaction With Life Scale, Subjective Happiness Scale	N/A	Gender dysphoria and body image difficulties persisted among transgender adolescents despite puberty suppression but remitted after cross-sex hormone therapy and gender reassignment surgery, with transgender women reporting more satisfaction with primary sex characteristics than transgender men	Netherlands
Dharma et al. [42]	N = 323	Body image	171 transmasculine participants and 152 transfeminine participants Adults, racial diversity present	Cross-sectional	Trans-Specific Condom/Barrier Negotiation Self-Efficacy adapted from the Self-Efficacy for Negotiating Condom Use Scale; Trans-Specific Sexual Body Image Worries created by the authors; Rosenberg Self-Esteem Scale; Center for Epidemiologic Studies Depression Scale; Multidimensional Sexual Self-Concept Questionnaire; Experiences of Transphobia; Sexual Risk of AIDS	N/A	T-Barrier was significantly associated with higher self-esteem and sexual satisfaction, and with lower sexual anxiety, sexual fear, and experiences of transphobia; however, there were no significant differences in T-Barrier scores by HIV risk, past year sexual partners, and gender spectrum. T-Worries had a positive correlation with sexual anxiety, sexual fear, and depressive symptomology, as well as a negative correlation with self-esteem	Canada
Duffy et al. [43]	N = 84	Eating Disorder	6 women, 30 men, and 48 nonbinary participants Adults, racial diversity present	Qualitative survey data	Demographics	Online qualitative questionnaire addressing psychiatric history, ED and treatment history, and experiences as a transgender person	One identified theme of this study regards the role of the body in eating disorder treatment, including the role of the physical body as the cause of the eating disorder, which was highlighted by about one third of participants. Others explained that the issue of body image is more complex for transgender clients and therefore eating disorder treatment needs to focus on more than a positive body image, and rather include transition in the eating disorder recovery process	United States, Europe, and Canada
Fagan et al. [44]	N = 66	Body Image	21 transgender individuals and 45 cisgender male participants Adults, Caucasian only	Cross-sectional	10 scales of the Sexual Functioning Inventory: Information, Experience, Drive, Attitude, Symptoms, Affects, Role, Fantasy, Body image, and Sexual satisfaction; The Brief Symptom Inventory; History of psychiatric diagnoses and sexual history	N/A	Trans participants were significantly more psychologically distressed than cisgender controls, with more negative affects consistent with dysphoric mood and other psychological symptoms. Trans participants also had a poorer body image, particularly with relation to sexual body parts	United States
Ferrucci et al. [45]	N = 10,415	Eating Disorder	10,415 transgender people. About 59% of the sample was described as female on insurance claims	Cross-sectional	ICD 10 codes for eating disorders, Covariates included age, region of medical service within the United States, relationship to plan-holder, sex reported on claims, and type of insurance coverage	N/A	Unspecified feeding and eating disorders were the most commonly diagnosed eating disorders, followed by anorexia nervosa, other specified feeding and eating disorders, bulimia nervosa, binge eating disorder, and avoidant restrictive feeding and intake disorder. Those diagnosed with any eating disorder were more likely to be young, reported as female on claims	
Feusner et al. [46]	N = 56	Body Image	16 trans men participants, 20 cisgender male controls, and 20 cisgender female controls Adults	Cross-sectional	Unmorphed own body image (degree that the image represents self), morphed body image (degree that the image represents self), response time	N/A	Trans men participants rated their bodies morphed to the non-natal sex higher compared to the natal sex, whereas female and male controls rated their morphed bodies lower when morphed to non-natal sex compared to natal sex. The transgender participants were generally more likely to rate the image as ‘‘self’’ when it was morphed with the non-natal sex, while the opposite was true for control participants	Sweden
Feusner et al. [47]	N = 81	Body Image	27 trans men participants, 27 cisgender male controls, and 27 cisgender female controls Adults	Cross-sectional	Body morph test; fMRI and resting state image data; the Kinsey scale; the Social Responsiveness Scale	N/A	The transgender individuals demonstrated weaker brain connections in areas related to self-referential processes and own body perception compared to control participants. Transgender participants were more likely to rate an image as ‘self’ when morphed with sex congruent with their gender identity, which was associated with greater brain connectivity in the areas studied	Sweden
Finn and Dell [48]	N = 7	Eating Disorder	3 assigned male at birth and 4 assigned female at birth Adults	Qualitative interviews	demographic data	No structured interview format was used	Themes described the pathological body as unhealthily `visible'; the relocation of gender re-embodiment and management from the discursive realm of health and distress to that of `choice'; and the productivity of choice in terms of the non-distressed experiences and subjectivities of those who choose to manage their bodies as differently gendered	United Kingdom
Fisher et al. [49]	N = 125	Body Image	66 trans women patients and 59 trans men patients Adults	Cross-sectional	Cross-sex hormone therapy use and dosage; levels of psychopathological distress by Italian version of the Symptom Checklist; the General Severity Index; the Body Uneasiness Test	N/A	Individuals taking CHT had significantly lower BUT-GSI scores, indicating lower body uneasiness, compared to those not taking CHT. Further, trans women participants demonstrated lower body uneasiness when taking CHT compared to those not taking CHT, but this difference was not found among trans men participants	Italy
Fisher et al. [50]	Cross-sectional study: N = 359 Cohort study: N = 54	Body Image	140 trans men and 219 trans women participants for cross-sectional analysis. The cohort study then included 28 trans women and 26 trans men participants Adults	Cross-sectional, then prospective cohort	Height, weight, BMI, testis volume via Prader Orchidometer, breast development according to Tanner staging, hair growth evaluated by Ferriman and Gallwey scoring system, clitoral length, glutamic-oxaloacetic transaminase and glutamic-pyruvate transaminase blood levels, Body Uneasiness Test, Symptoms Checklist 90 Revised, Gender Identity/Gender Dysphoria questionnaire, Beck Depression Inventory II	N/A	Transgender participants taking CHT reported significantly lower levels of body uneasiness, depressive symptoms, and subjective gender dysphoria compared to those not taking CHT. CHT-induced body modifications, such as changes in hair growth, testis volume, and clitoral length, were associated with better psychological adjustment	Italy
Gleming et al. [51]	N = 44	Body Image	22 trans men and 22 cisgender matched control males Adults	Cross-sectional	Body Cathexis Scale, 10-item version of Janis-Field-Eagley Self-Esteem Measure	N/A	Significant association between increased body satisfaction and increased amount of surgical gender reassignment. Moderate associations were found between a positive body image and high self-esteem for the no-hysterectomy and hysterectomy groups, while a strong associated was found for the phalloplasty group	Not reported, likely United States based on author location
Gagne and Tewksbury [52]	N = 65	Body image	27 preoperative transgender individuals, 10 postoperative transgender individuals, 4 nonoperative transgender individuals, 19 crossdressers, and 5 gender radicals Adults, racial diversity present	Qualitative	None	Interview topic areas included background information, life stories and experiences of being transgender, and political and gender attitudes	Individuals commonly reported beginning to think of their penises as "birth defects," their bodies as false signifiers of who they really were, and their alternatively gendered selves as a medical condition in need of a cure for creating an accepted and embodied self	United States
Hartman-Munick et al. [53]	N = 98	Eating disorder	24 transgender women, 40 transgender men, 27 nonbinary adults, and 6 who identified as another gender. Racial diversity present	Qualitative, Inductive	N/A	online forum and focus group questions about experiences with ED	Three major themes emerged from the analysis: (1) Barriers to ED screening/treatment; (2) Complexity of the relationship between EDs and gender dysphoria; (3) Need for provider education in gender affirming care practices for ED screening and treatment Results indicate an ongoing need for gender affirming care for TGD young adults in ED screening and treatment	United States
Hepp and Milos [54]	N = 3	Eating disorder	3 adult transgender patients	Case series	Demographics	Relevant medical history	Transgender individuals may have increased risk for eating disorders	Switzerland
Hiraide et al. [55]	N = 2	Eating disorder	1 trans woman individual and 1 trans man individual. Both adults	Case report	Demographics and BMI	Relevant medical history	When gender role conflict is persistent, maladaptive eating behaviors could be worsened. Gender affirming medical intervention may alleviate eating disorders	Japan
Isung et al. [56]	N = 10	Body image	10 trans women adults	Prospective cohort study of the impact of surgical treatment	Transgender Congruence Scale, Body Image Scale, Hospital Anxiety and Depression Scale, Sheehan Disability Scale, EuroQol-5-Dimensions visual analogue scale, age	None	Appearance congruence and body image satisfaction improved by gender-affirming surgery	Sweden
Jones, et al. [18]	n = 563	Body image and eating disorder	211 people assigned female at birth and 352 people assigned male at birth Adults	Cross sectional	Eating Disorder Inventory 2, Rosenberg Self Esteem Scale, Hospital Anxiety and Depression Scale	N/A	Transgender people who were not on cross-sex hormones reported higher levels of eating disorder psychopathology than people who were	United Kingdom
Joy et al. [57]	N = 7	Eating Disorder	7 gender diverse participants	Qualitative	N/A	ED treatment experiences	Four themes around gender dysphoria were constructed from the data, including gender dysphoria and eating disorders, barriers to accessing eating disorder treatments, harmful eating disorder treatment strategies and suggestions for eating disorder programmers and health professionals. Gender dysphoria considerations were believed to be lacking in traditional eating disorder treatment programs. Participants saw the need for more awareness and training in this area for dietitians and other health professionals	Canada
Khoosal et al. [58]	N = 40	Body image and eating disorder	40 trans women gender identity disorder patients Adults	Cross sectional	Eating Disorder Inventory	N/A	Concerns about body satisfaction, lower levels of drive for thinness, bulimia were greatly reduced after gender affirming transitioning/surgery	United Kingdom
Kilpatrick et al. [59]	N = 83	Body image	40 transgender men, 24 transgender women, and 19 controls Adults	Cohort	Body perception test (mophed body image testing), cortical thickness, MRI data, Kinsey scale; Demographic information	N/A	Cross sex hormone therapy reduces gender dysphoria evidenced by patient report and pattern changes in the cerebral grey	Sweden
Kraemer et al. [60]	N = 45	Body image	23 preoperative and 22 postoperative transgender patients Gender Identity Trans women: 30, Trans men: 15	Cohort	Body Image Measure, which consisted of the insecurity/concern scale, the attractiveness/self confidence scale, and the accentuation of body appearance scale; Demographic information	N/A	Trans women scores on measures were similar to cisgender females and trans men scores more like cisgender males (pre and post-operative). Trans women and trans men body image scores changed as expected, with increased positive body image post-gender affirming surgery	Germany
Linsenmeyer et al. [61]	N = 10	Eating disorder	10 transgender men	Case series	The EAT-26 and ecSI-2 measures were utilized	Three-day food diary and the software ESHA Food Processor Nutrition Analysis	Major nutrition-related concerns were obesity, low fruit and vegetable intake and high sodium intake; disordered eating was not a prominent concern. None fo the participants screened positive for an ED	United States
Majid et al. [62]	N = 60	Body image	30 transgender individuals and, 30 cisgender individuals	Cross-sectional	FMRI, Body perception task	N/A	Transgender participants consistently reported non-birth sex bodies looked more like "me."	Sweden
Mitchell et al. [63]	N = 130	Body image and eating disorder	41 transgender women, 42 transgender men, 47 nonbinary individuals	Cross-sectional	Misgendering frequency, transgender congruence, Body Parts Satisfaction Scale Revised, Disorder Examination-Questionnaire (EDE-Q), 10 transition steps they had undertaken	N/A	The overall sample showed significant direct effects between misgendering frequency and transgender congruence, between transgender congruence and body dissatisfaction, and between body dissatisfaction and dietary restraint. There was a significant indirect effect between misgendering frequency and body dissatisfaction through transgender congruence, and between misgendering frequency and dietary restraint through both transgender congruence and body dissatisfaction (but not through either mediator alone)	United States
Morgan and Stevens [64]	N = 6	Mind–body dissonance	5 trans women individuals and one participant identified as a crossdresser. Adults, all racially white	Qualitative	None	Semi-structure interview about gender identity development and body-mind dissonance (i.e., dysphoria)	Participants described early awareness of body-mind dissonance in early childhood. Most described waiting to do a gender transition for varied reasons	United States
Nagata et al. [65]	N = 484	Eating disorder	312 transgender men and 172 transgender women	Cross-sectional	Eating Disorders Examination Questionnaire (EDE-Q)	N/A	Transgender men and women reported any occurrence (≥ 1/week) of dietary restraint (25.0% and 27.9%), objective binge episodes (11.2% and 12.8%), excessive exercise (8.0% and 8.1%), self-induced vomiting (1.6% and 1.7%), and laxative misuse (.3% and .6%), respectively. Compared to a prior studies, our age-matched subsample of transgender men reported lower rates of objective binge episodes and excessive exercise and transgender women reported higher rates of dietary restraint but lower rates of excessive exercise	United States
Nikkelen and Kreukels [66]	N = 576	Body image	325 trans women and 251 trans men transgender people Age diversity present	Cross sectional	Kessler Psychological Distress Scale, scale constructed by study about body image	N/A	Gender-affirming treatment can positively affect sexual feelings of desire, particularly in trans women, but body satisfaction may be a more significant factor driving sexual desire	Netherlands
Nowaskie et al. [67]	N = 166	Eating disorder	79 transgender men and 87 transgender women	Cross sectional	Eating Disorder Examination Questionnaire (EDE- Q), demographics	N/A	Compared to transgender men, transgender women reported higher EDE-Q scores and significantly higher Eating Concern. Compared to hormone/surgery-naïve and hormone-experienced/surgery-naïve patients, hormone/ surgery-experienced patients had lower EDE-Q scores. Hormone/surgery- experienced patients reported significantly lower Shape Concern and marginally lower Global Score and Weight Concern than hormone-experienced/surgery-naïve patients. There were no differences in EDE-Q scores between hormone/surgery- naïve and hormone-experienced/surgery-naïve patients	United States
Owen-Smith et al. [11]	N = 697	Body image	347 TM individuals and 350 TF individuals Adults, racial diversity present	Cross sectional	Transgender Congruence Scale, Revised Physical Self-Perception Profile, Center for Epidemiologic Studies Depression Scale, Beck Anxiety Index	N/A	More extensive gender-affirming treatment was associated with higher body-gender congruence and body image satisfaction, and lower depression and anxiety	United States
Rabito Alcón et al. [68]	N = 52	Body image and eating disorder	30 trans woman and 22 trans man Adult	Cross-sectional	Eating Attitudes Test, body dissatisfaction of Eating Disorder Inventory-2 sub-scale, and IMAGEN	N/A	Body dissatisfaction was associated with thinness in people with gender dysphoria. The level of body dissatisfaction associated with thinness is above the general population in people with gender dysphoria	Spain
Simbar et al. [69]	N = 90	Body Image	31% female participants and 69% male participants Patients with no hormone therapy or surgery: 30, Patients with hormone therapy: 30, Patients with gender reassignment surgery: 30, Adults	Cross sectional	Quality of Life questionnaire and Fisher's Body Image questionnaire	N/A	Surgery significantly improved the quality of life and body image of individuals with gender dysphoria. Body image, quality of life, and physical health were all positively correlated. Authors highlighted the significant social health on the quality of life scale	Iran
Testa et al. [70]	N = 442	Body Image and eating disorder	154 transfeminine people and 288 transmasculine people Adults, racial diversity present	Cross-sectional	Non-affirmation of gender identity scale, body areas satisfaction scale, Eating Attitudes Test; Gender-Confirming Medical Interventions planning/completed questionnaire	N/A	No significant differences in eating attitudes between those who had and had not completed gender affirming medical interventions. Generally, participants who had surgery and hormone use had more body satisfaction than those who did not have these procedures	United States
Turan et al. [71]	N = 1	Eating disorder	One 41-year-old trans man	Case study	None	N/A	Being underweight enabled the suppression of menstruation and female secondary sexual characteristics, with the goal of rejecting femininity rather than to look slim. Sex reassignment surgery greatly alleviated eating disorder symptoms	Turkey
Turan et al. [72]	N = 77	Body image and eating disorder	Trans man Participants: 37 Female control participants: 40 Adults	Cross-sectional	Body Uneasiness Test, Eating Attitudes Test Symptom Checklist-90-Revised, (90-item self-report inventory that measures ten symptoms of psychopathology- Somatization, Obsessive–Compulsive, Interpersonal Sensitivity, Depression, Anxiety, Hostility, Phobic Anxiety, Paranoid Ideation, Psychoticism, and Additional over a 1-week interval	N/A	Compared to baseline, after 24 weeks of cross hormone therapy participants had a decrease in body uneasiness and general psychopathological symptoms, but no difference in eating attitudes or behaviors. There was an increase in mean body weight and BMI scores	Turkey
Uniacke et al. [73]	N = 287	Eating disorder	116 transgender women, 93 transgender men, 78 nonbinary people	Cross sectional	Eating Disorder Examination; Transgender Congruence Scale (TCS); Everyday Discrimination Scale; Brief Symptom Inventory	Data were collected using structured interviews conducted by trained interviewers in each city	Higher transgender congruence was associated with lower odds of disordered eating symptoms, whereas increased internalized transphobia (minority stress) was associated with greater odds of disordered eating symptoms. Participants with eating-related psychopathology had greater odds of having received gender-affirming psychotherapy in the year prior to assessment—comorbidity or reason to seek gender affirming medical intervention	United States
van de Grift et al. [74]	N = 485	Body image	Transgender adult men and women	Cross-sectional	Body Image Scale for transsexuals; Demographic data, information on social gender role, and previous medical treatment were taken from a standardized self-constructed background interview	N/A	Trans women participants reported strongest dissatisfaction with genitals, breasts, and social and hair growth items. The trans men respondents reported that they were most dissatisfied with their chests and genital body characteristics	Netherlands, Belgium, Germany, and Norway
van de Grift et al. [75]	N = 660	Body Image	Transgender adult men and women	Cross-sectional	Body Image Scale, Physical Appearance Scale; Gender identity disorder diagnosis and sexual orientation measured by one item from a semi-structured background interview	N/A	Trans women indicated lower body satisfaction and a less congruent physical appearance with their experienced gender than trans men. Participants with prior hormone treatment had significantly higher physical congruence with experienced gender than those without prior hormone use	Netherlands, Belgium, Germany, and Norway
van de Grift et al. [76]	N = 201	Body Image	Adults transgender sample	Retrospective cohort, then cross sectional	Utrecht Gender Dysphoria Scale, Body Image scale, Symptom Checklist 90, Satisfaction With Life Scale, the Subjective Happiness Scale, and the Cantril Ladder, Multidimensional Sexuality Questionnaire, Social Support Questionnaire	N/A	Improvements in body satisfaction followed hormone-based interventions and surgery. There was a significant association between body satisfaction at follow-up and the level of psychological symptoms and the degree of body satisfaction at baseline	Netherlands, Belgium, Germany, and Norway
van de Grift et al. [77]	N = 101	Body image	Adult transgender men	Cross-sectional	BODY-Q scores, Postoperative patients were asked: "Do you plan to apply for a secondary correction of your chest? (yes/unsure/no)" and "Do you experience feelings of anxiety/depression?"	N/A	Mastectomy had a positive effect on patient-reported satisfaction with appearance, health-related quality of life, and psychological function. A higher body mass index was associated with lower body satisfaction in both pre- and postoperative patients	Netherlands
van de Grift et al. [78]	N = 26	Body image	Adult transgender men sample	Prospective follow-up cohort	Appearance Schemas Inventory-Revised, Body Image Quality of Life Inventory, Body Image scale for Transsexuals, Multidimensional Body-Self Relations Questionnaire, Rosenbergy Self-Esteem Scale, Situational Inventory of Body-Image Dysphoria, Author developed Perceived Effect of Surgery scale	N/A	Mastectomy positively associated with body image. Positive evaluation of the body was associated with increased quality of life and self-esteem, as was decreased dysphoria during social situations	Netherlands
Velez et al. [79]	N = 304	Body image and eating disorder	Adults, racial diversity present	Cross-sectional	Heterosexist Harassment rejection and discrimination scale, Transgender congruence, Sociocultural attitudes toward attractiveness questionnaire-3, Body surveillance, Body satisfaction, Compulsive exercise	None	Internalization of sociocultural standards of attractiveness yielded a significant direct relation with compulsive exercise, and anti-transgender discrimination	United States
Vocks et al. [80]	N = 356	Body image and Eating disorder	43 trans men, 88 trans women 62 cisgender women with eating disorders, 56 control cisgender males, 107 control cisgender females. Adults	Cross-sectional	Eating Disorder Examination Questionnaire, Eating Disorder Inventory, Body Checking Questionnaire, Drive for Muscularity Scale, Rosenberg Self-Esteem Scale, Beck Depression Inventory	N/A	Trans men and trans women participants showed higher depression scores, restrained eating, weight concerns, shape concerns, body dissatisfaction, and body checking compared to controls. Between trans women and trans men participants, body checking was the only significant difference, as trans women displayed higher scores	Germany, Austria, and Switzerland
Watson et al. [81]	N = 923	Eating disorder	Adolescent sample, age and racial diversity present	Cross-sectional	School connectedness, family connectedness, perception of friends caring, Medical outcomes study social support survey, Questions regarding binge eating, lose weight by fasting/diet pills/laxatives/and vomiting	N/A	Higher rates of harassment and discrimination was linked to higher odds of disordered eating behavior such as binge eating, fasting, or vomiting to lose weight, while family and school connectedness and social support had a protective effective against odds of disordered eating	Canada
Weyers et al. [82]	N = 50	Body image	Adult sample of trasngender women	Cross-sectional	Dutch version of the Short-Form-36, visual analog scale, Dutch version of the Female Sexual Function Index, additional questions about medical history, quality of relationships, importance of sex, concerns about health, and regret concern gender transition	N/A	Results showed a connection between psychological and sexual functioning in trans women, specifically with difficulties around arousal, lubrication, and pain	Belgium
Winston et al. [83]	N = 2	Eating disorders	Two transgender people assigned male at birth	Case study	None	N/A	In both cases, the desire for thinness was associated with a wish to achieve a more feminine physique. Both patients had educational difficulties	United Kingdom
Witcomb et al. [84]	N = 600	Body image and eating disorder behavior	75 transgender males, 125 transgender females, 200 cisgender individuals with eating disorders Adults	Cross-sectional	Eating disorder inventory 2, Hamburg body drawing scale	N/A	Those with eating disorders, cisgender females, and transgender females scored higher overall on drive for thinness. The eating disorder group had significantly higher body dissatisfaction scores than both the trans group, which had higher scores than the control group	United Kingdom
Wolfradt and Neumann [85]	N = 90	Body image	30 post-operative transgender women, 30 cisgender women, and 30 cisgender men Adults	Cross sectional	German versions of questionnaires, including scales for depersonalization experiences, the Self-Esteem-Scale, the Body-Image Questionnaire, a Gender Identity Trait Scale, and life satisfaction	N/A	Depersonalization decreased after gender affirming surgery. The transgender participants had body attitudes that were more similar to cisgender males, but described themselves with more feminine traits	Germany

After the review process, these are the final articles retained for review and used in results summaries

**Table 2:** Synthesis of results based on review questions

Review Question	Summary of Results
What are the risks and protective factors for eating and body image related problems?	Risks include dehumanization, objectification, and discrimination, hyper-femininity, societal cultural femininity and masculinity ideals, high body dissatisfaction, misgendering, perfectionism, anxiety symptoms, and low self-esteem Protective factors include receiving gender-affirming medical intervention and social support
Who is being included and excluded in the TGNB samples of studies on eating and body image related problems?	Predominately racially white samples Lack of nonbinary participants Language variation about transgender identities and diagnoses
What are the empirically supported treatments for eating and body image problems for TGNB patients?	No single treatment modality has been tested in a clinical trial. Most treatment studies were case studies Gender affirming medical intervention was used alongside other treatment modalities for alleviating eating disorder symptomology, gender dysphoria, and body image problems Cultural adaptation of eating disorder treatment modalities to fit the needs of TGNB patients TGNB adults experience barriers to accessing treatment

### Bias and limitations

Review of bias and limitations across studies showed five studies lacked limitations sections and 10 studies were missing acknowledgments of potential biases in the study. Many of the studies lacked a full description of the methodology and demographics of the sample. Over 78% of studies (*n* = 47) did not include a guiding theory. Of the studies that did include theory, MST (*n* = 5; 8.5%) [6] and objectification theory (*n* = 4; 6.8%) [86] were the most commonly identified. In two cases, the studies drew on both MST and objectification theory. Most studies were not funded. Only 18 were funded from a mix of funders—federal or national sources (e.g., Dutch Ministry of Health; National Institute for Health) and internal university or medical system funding (e.g., Harvard Catalyst).

Some studies defined “transgender” and delineated non-binary or gender queer individuals in the samples. Where possible, non-binary and gender queer samples were analyzed in the results separately. Given the clinical nature of many of the studies and samples, some relied on the existence of gender dysphoria or the older diagnosis of gender identity disorder (*n* = 22). Three studies defined, and some diagnosed, transsexualism (e.g., Kirkpatrick et al., 2019; Kraemer, et al., 2008) now seen as outdated and offensive language. These studies seemed to be grounded in the diagnostic criteria and current language and clinical thinking about transgender identities. While other studies used less clinical terms, like transgender, gender minority, or gender nonconformity or incongruence and, in some cases, allowed for self-identification as part of the inclusion criteria. Some of the clinical samples lacking explicit definition of transgender denoted “male-to-female” or “female-to-male” gender transitions (*n* = 10) were in progress, complete, or the person was seeking gender affirming clinical medical care. Given the dated and potentially offensive language used in some of the studies, the results, discussion, and tables (see Tables 1 and 2), this article uses the terms transgender, trans men/women, and nonbinary where appropriate.

### Method analysis

#### Quantitative findings

A total of 48 studies relied on quantitative measurements, scales, and analyses. Most of the articles retained for this study employed a cross-sectional study design (*n* = 46). Several different measures were used to assess the variables of interest, with the most common being the Eating Attitudes Test, Body Uneasiness Test, Symptoms Checklist-90, and Eating Disorders Inventory. The sample sizes of the quantitative studies were well-distributed from less than 50 to over 5000. Demographically, most quantitative studies did not report racial data as part of the sample characteristics, and of the studies that did report racial information, 14 included primarily racially White samples. Most of the quantitative studies originated from outside of the United States, including from Italy (*n* = 8), Sweden (*n* = 5), UK/England (*n* = 2), multicenter data from four European countries (*n* = 5), Germany (*n* = 4), Netherlands (*n* = 2), Belgium (*n* = 1), Turkey (*n* = 1), Iran (*n* = 1), Spain (*n* = 1), Canada (n = 1), Finland (*n* = 1), multicenter data from the United States, Canada, and Europe (*n* = 1). The remaining 15 quantitative studies included data collected only in the United States.

Two studies included both a cohort and cross-sectional element [50, 76]. Both studies assessed body image variables and focused on adult populations. Both studies included European samples; one included patients at a clinic in Italy [50], and the other included patients at gender clinics in the Netherlands, Belgium, and Germany [76]. The gender affirming interventions analyzed in these studies included hormone replacement therapy and gender-affirming surgeries. Six studies included in our analysis utilized a cohort design [41, 56, 59, 60, 76, 78]. All the cohort studies examined body image and most used sample sizes under 50 participants [41, 56, 59, 60]. Half of the cohort studies analyzed the impacts of surgical interventions [56, 60, 76], while others studied hormone replacement therapy [59] or a combination of hormonal and surgical gender-affirming interventions [41, 76]. A variety of measures for eating disorders and body image were used with very little overlap between studies. Two studies were conducted in the Netherlands, two were conducted in Sweden, and one was conducted in Switzerland.

#### Qualitative and case report studies

Within the articles retained for this scoping literature review of TGNB adults, 12 utilized a qualitative or case study approach. The case studies (*n* = 5) detailing one to five individual cases of gender dysphoria, eating disorders, body image and dissatisfaction, and obesity with quantitative and qualitative data presented. The findings from these case reports focused on psychiatric care [54] and eating disorder treatment [83]. Two case reports included detailed patient medical records [55, 71]. Four qualitative studies interviewed transgender people [48, 64, 87, 88]. Three of the interview articles included large qualitative sample sizes ranging from 30 to 98 transgender participants. Demographics of the samples in qualitative studies were younger with most being under age 35. Race and ethnicity were not always offered in the published article, though when reported was predominately racially White in samples from the United States. The interview articles with larger samples showed greater age and racial/ethnic diversity.

### Eating disorders and treatment

Existing evidence related to eating patterns among the TGNB population centers on the prevalence, risk, and protective factors for eating disorders and disordered eating. In addition, many articles considered treatment outcomes and other clinical concerns for individuals who have been diagnosed with an eating disorder.

#### Eating disorders

Estimates of eating disorder and disordered eating prevalence among TGNB adults used surveys with smaller population samples [38] and medical records [45]. Medical records data from the United States found that among over 10,000 TGNB patients, between 0.15 and 1.37% were diagnosed a variety of feeding and eating disorders [45]. The prevalence studies sometimes grouped transgender participants into one category or further subdivided into trans man or trans woman identities in others. Most studies utilized validated measures to assess eating disorder risk including: the Eating Attitudes Test-26 (EAT-26) [4, 35, 40], the Eating Disorder Inventory (EDI) [28, 38, 84, 89], and the Eating Disorder Examination Questionnaire (EDE-Q). Others used select questions related to disordered eating patterns [29]. Two measures were tested for validity and reliability with TGNB adults—the EDE-Q and the Nine-Item Avoidant/Restrictive Food Intake Disorder Screen (NIAS). Nagata et al. [65] established community norms for the EDE-Q among adult transgender men, transgender women, and gender expansive individuals [2].

#### Correlates and risk factors

The existing body of research also addressed correlates, risk, and protective factors for eating disorders and disordered eating among the TGNB population. Correlations of disordered eating with other constructs were reported such as dehumanization, objectification, and discrimination [4], hyper-femininity [35], societal femininity ideals, and a feminine gender role orientation [38]. Vocks et al. (2009) reported differences in eating disturbances among those with gender identity disorder where transgender women reported higher restrained eating, eating concerns, weight concerns, shape concerns, drive for thinness, bulimia, body dissatisfaction, and body checking compared to cisgender women [80]. Transgender men reported higher restrained eating, weight concerns, shape concerns, body dissatisfaction, and body checking compared to cisgender men. Risk and for eating disorders were estimated from clinical survey data finding high body dissatisfaction, perfectionism, anxiety symptoms, low self-esteem [18].

#### Eating disorder treatment

Authors discussed approaches and treatment considerations for TGNB patients with eating disorders. Treatment considerations included management of coexisting mental health diagnoses [90] and the complex interplay between eating disorder psychopathology and one’s gender identity or gender dysphoria for adults [28, 54, 72]. For example, Hepp and Milos (2002) discussed the relationship between eating behaviors, gender identity, sexual orientation and body dissatisfaction using three case studies of transgender patients diagnosed with an eating disorder [54]. Ålgars et al. (2012) used semi-structured interviews to explore participants own understanding of the causes of their disordered eating; most participants reported experiences with disordered eating that were attributed to a desire to bring their bodies into alignment with their gender identity [28].

Studies on eating disorder treatment also addressed the impact of gender-affirming medical interventions on eating disorder symptomology or recovery using case studies [55, 91, 92] and clinical or survey data [18, 70, 71, 89]. Medical interventions included masculinizing or feminizing hormone therapy [18, 70, 71] and gender-affirming surgeries [55, 70, 71, 89]. Most of the evidence demonstrates eating disorder symptomology improves, but does not entirely resolve, with gender-affirming medical interventions. On the other hand, Turan et al. (2018) found that eating attitudes and behaviors did not significantly improve among transgender men after six months of hormone therapy [72]. Overall, authors of the existing research emphasized that although access to gender-affirming medical interventions may be critical to address the underlying cause, treatment of the eating disorder is still warranted.

Lastly, only two studies centered on the lived experiences of TGNB adults in eating disorder treatment. The studies described the role of the body in eating disorder treatment, negative experiences with clinicians, recommendations for treatment centers and providers caring for TGNB patients with eating disorders, the importance of centering the lived expertise of TGNB people about their experiences and needs [53], and barriers to accessing competent care [57]. Both emphasized the need to address gender dysphoria in eating disorder treatment [53, 57].

### Body image—satisfaction, shame, and surveillance

Most studies (*n* = 44) examined a body image construct either as a primary finding or alongside eating disorders. Body image, as it applies to TGNB populations, utilized varied construct definitions leading to a variety of measures for body image including assessing body satisfaction, shame, and surveillance. Body shame referred to the perception of one’s body failing to meet cultural expectations of attractiveness that become internalized through objectification experiences [40]. Body surveillance, the constant and persistent monitoring of the body, was utilized in just three studies alongside other body image and eating disorder measures [4, 40, 79] for testing objectification theory. Some of the measures utilized included the Body Image Assessment Questionnaire (FBeK), the Body Cathexis Scale (BCS), Body Image Scale, Lindgren-Pauly Body Image Scale, Body Uneasiness Test, Body Dissatisfaction, and Fishers Body Image questionnaire.

Misgendering, or being referred to by the incorrect name, pronouns, or other language that does not align with one’s gender identity, was associated with decreased body satisfaction and restricted eating [63]. Negative body image was associated with altered eating patterns in two studies [40, 93]. Comiskey et al. (2020) found body shame was positively associated with disordered eating and the intention to alter one’s body due to the internalization of cultural standards of beauty for transgender women [40].

Body image was also measured to determine if treatment targeting body image could alleviate gender dysphoria and improve body image and mental health. Eight studies utilized body image as a variable with TGNB samples and included diagnosed conditions of body dysmorphia, gender incongruence, or gender identity disorder [9, 30, 32, 42, 46, 51, 56, 60]. Treatment considerations for TGNB individuals included clinical interventions of psychotherapy, gender affirming hormone therapy, medication, and genital/chest surgery [30, 51, 74, 78]. Those who received surgery reported improved body image and body satisfaction after the top and bottom surgery [76, 77, 89]. Those who received top surgery reported higher body satisfaction and psychological functioning second to improved body and gender identity congruence [77].

## Discussion

Existing evidence supports the TGNB adult population experiences elevated rates of eating disorders and disordered eating when compared to cisgender populations [29]. There is, however, an inherent limitation to estimating eating disorder and disordered eating prevalence in that only an initial study offered measurement validity and reliability for use with TGNB adults [2]. TGNB individuals may utilize eating or exercise behaviors for purposes distinct from the cisgender population such as weight manipulation for a body size or shape that better aligns with one’s gender identity, pubertal and/or menstrual suppression, and masking of body features that do not align with one’s gender identity [61]. This creates a complex and common intersection of gender dysphoria, body image, and eating patterns for TGNB people [53, 57]. Despite recommendations to screen TGNB patients for eating disorders, existing measures on eating patterns may not be accurate or reliable with TGNB [2]. Future research should continue to validate existing measures for use among the TGNB individual from minoritized racial/ethnic groups, adapt existing measures to address considerations specific to TGNB patients who seek treatment, or develop and validate new measures.

The risk and protective factors identified in MST associated with eating disorders and body image problems across four decades of studies suggests gender-affirming medical interventions (i.e., gender affirming hormone therapy and surgeries) may alleviate eating disorder symptomology and negative body image [9, 11, 18, 31, 41, 49–51, 55, 56, 59, 60, 67, 69, 71, 72, 75–78, 85, 89]. Though the degree of social stigma [63], cultural standards of femininity and masculinity [35, 40, 79], internalized transphobia [73], lack of social support and acceptance [81], childhood trauma [32], and discrimination [4, 79, 81] experienced by TGNB adults also impacted symptomology limiting the impact of gender-affirming medical interventions alone. Therefore, traditional eating disorder treatment may still be warranted alongside gender-affirming medicine. Furthermore, additional treatment for addressing these minority stressors and promoting a positive body image for TGNB people that goes beyond stereotypical cultural standards are warranted [43].

The existing research is primarily observational in nature. Little is known about how TGNB patients may respond to eating disorder prevention or treatment approaches traditionally designed for cisgender populations. Riddle and colleagues responded that TGNB patients may show improved symptomology in higher levels of eating disorder treatment yet may experience more severe depression and show less improvement during treatment compared to cisgender patients [1]. In contrast, Duffy and colleagues reported that eating disorder treatment may be ineffective from the perspectives of TGNB adults and cause harm to due lack of clinician preparedness or mistreatment from healthcare providers [43]. Future research may explore the efficacy of eating disorder prevention and treatment approaches that are culturally adapted for TGNB adults, treatment for concurrent mental health conditions, as well as approaches to training healthcare professionals and students to provide competent care for TGNB adults. In this effort, MST could be introduced as a guiding theory for considering the social and relational context of discrimination, gender body norms, and structural barriers to health and healthcare.

The review found variations and absences in definitions of transgender, body image or shame, and guiding theories. This demonstrates the changing language, diagnostic criteria, emergence of MST over the past 40 years, and clinical conceptualizations of eating and body image as uniquely intertwined among TGNB populations. The studies rarely addressed social and interpersonal factors; either through theory or inquiry (e.g., including questions about family, relationships, employment, etc.) despite the significance of social supports and resources on the health of TGNB people demonstrated in MST. These social needs are lifelong for TGNB adults [94]. Future research that is inclusive of social factors for understanding eating patterns and body image would be well guided by MST for considering relational dependencies [95], housing stability, and food insecurity [96] that significantly shape eating patterns and health.

Many of the studies, especially qualitative studies, lacked racial and ethnic diversity in the samples and none looked at non-binary or gender queer individuals exclusively. This may reflect high concealment and the associated risk of violence experienced by TGNB people from minoritized racial groups [97] and observations of increased racially white, female-assigned at birth TGNB individuals accessing gender-affirming medical care in some areas of the United States [98]. In case reports and reviews with small sample sizes and known locations, demographic information and the stories of the patients increase the risk for making an already vulnerable TGNB person identifiable. Although case reports are common practice in medical sciences for exploring new phenomena and encouraging emergent treatment, it raises ethical concerns for use of single or identifiable patient records when there are known risks of discrimination and violence.

In addition, the purpose of the study, even in large sample sizes, may begin with a bias where individual academic medical professionals who are not utilizing MST as a framework for understanding the social determinants of health of TGNB people, could further pathologize TGNB patients in their care based on weight and eating patterns [99]. Therefore, future research may explore eating and body image among racially and ethnically marginalized groups of TGNB adults grounded in MST for identifying empirically known factors with a community-participatory action approach to methodology [100] for aiding in expanding and critiquing MST. The critique could draw from intersectionality for illuminating the experiences of those minoritized both racially and in terms of gender expression and identity [101]. Community-participatory designs can center the voices of TGNB people from racially and ethnically marginalized groups while paying for lived expertise as part of the research team. Successful use of this method has informed improved healthcare systems [102] and clinical education with TGNB adults [103]. Similarly, future research should center non-binary or genderqueer populations and their experiences through initial qualitative inquiry aimed at identifying potential cultural adaptations to eating disorder treatment and mental health services.

## Limitations

This review has several limitations. The team carefully planned and utilized software to accurately answer the study questions and conduct the review. However, research studies may have been missed. The current studies still lack nuance by variations in gender identity, expression, and social factors. Limiting the search by English means other international studies were missed. Some of the studies included transgender and non-transgender samples, requiring reliance on portions of the data or only descriptive analysis. Many of the studies were cross-sectional in nature, limiting causal associations between risk factors, treatments, and outcomes documented in the studies. Finally, the rigor could have been enhanced by pre-registering our search protocol with the International Prospective Register of Systemic Reviews.

## Conclusion

The scoping review offers an overview and examination of research with TGNB adults who experience eating and body image related problems as well as clinical studies on treatment approaches and effectiveness. The 59 studies identified demonstrated the significance of gender-affirming medical interventions for alleviating eating and body image issues though with limitations given the social stigma and discrimination experienced by TGNB people. Future research should consider the use of theory for guiding inclusion of salient social factors influencing eating patterns, body image, and treatment outcomes. In addition, more studies are needed with those from minoritized racial and ethnic groups and varied gender identities (e.g., nonbinary people) for identifying differences in needs and treatment modalities.

## Data Availability

Search terms and data retrieved through library searches are available upon request to the corresponding author. Data from searches is available upon request to the first author.
